# Post-Transplant Cardiovascular Disease in Kidney Transplant Recipients: Incidence, Risk Factors, and Outcomes in the Era of Modern Immunosuppression

**DOI:** 10.3390/jcm13102734

**Published:** 2024-05-07

**Authors:** Chukwuma Austin Chukwu, Anirudh Rao, Rachel Middleton, Philip A. Kalra

**Affiliations:** 1 Faculty of Biology, Medicine and Health, Division of Cardiovascular Medicine, The University of Manchester, Manchester M13 9PL, UK; anirudhrao@nhs.net (A.R.); rachel.middleton@nca.nhs.uk (R.M.); philip.kalra@nca.nhs.uk (P.A.K.); 2 Department of Nephrology, Salford Royal Hospital, Northern Care Alliance NHS Foundation Trust, Salford M6 8HD, UK; 3 Department of Nephrology, Royal Liverpool Hospital, Liverpool Hospitals NHS Foundation Trust, Liverpool L7 8YE, UK

**Keywords:** kidney transplantation, post-transplant cardiovascular disease, allograft survival

## Abstract

**Introduction**: Post-transplant cardiovascular disease (PTCVD) poses a significant challenge in kidney transplantation, potentially impacting graft outcomes and patient survival. This retrospective study aimed to investigate the incidence, risk factors, and consequential impact of PTCVD in kidney transplant recipients (KTRs) devoid of pre-existing cardiovascular disease (CVD). **Method**: The cohort comprised 1114 KTRs, with 749 individuals included after excluding those with pre-existing CVD and early graft loss. PTCVD encompasses ischemic heart disease, myocardial infarction, arrhythmias, heart failure, stroke, peripheral vascular disease, and valvular heart disease. Competing risk regression analysis was performed to identify predictors of PTCVD, while Cox proportional hazards analysis assessed the impact of PTCVD on graft and recipient survival. **Results**: The cumulative incidence of PTCVD at 5, 10, and 20 years was 5.4%, 14.3%, and 22.5%, respectively. Competing risk regression identified increased age (sub-hazard ratio [SHR], 1.22; *p* = 0.036) per decade, duration of dialysis (SHR, 1.07; *p* = 0.048) per year on dialysis, and the slope of the estimated glomerular filtration rate (SHR, 1.08; *p* = 0.008) mL/min/year decline as independent predictors of higher-risk PTCVD. A higher baseline estimated glomerular filtration rate (eGFR) was protective (SHR, 0.98; *p* = 0.032). PTCVD was not significantly associated with death-censored graft loss (adjusted hazard ratio [aHR] 1.31; *p* = 0.48) but was correlated with higher all-cause graft loss (aHR, 1.71; *p* = 0.011) and recipient mortality (aHR, 1.97; *p* = 0.004). **Conclusion**: This study provides insights into PTCVD predictors. Although not directly associated with graft loss, PTCVD significantly correlates with heightened mortality in kidney transplant recipients, emphasizing the need for enhanced clinical management and surveillance strategies.

## 1. Introduction

Cardiovascular diseases (CVD) pose a significant challenge in the aftermath of kidney transplantation, exerting a profound influence on both graft outcomes and patient survival. Despite notable advancements in immunosuppressive therapy, which have reduced acute rejection rates and improved short-term transplant outcomes, the trajectory of long-term allograft survival has remained relatively stagnant over the past two decades [1,2]. A critical factor contributing to this enduring challenge is the persistent burden of post-transplant cardiovascular disease (PTCVD), which becomes increasingly prevalent in kidney transplant recipients (KTRs) who experience extended lifespans. A substantial body of evidence consistently indicates a significantly higher incidence of cardiovascular disease among kidney transplant recipients compared to the general population [3,4]. Beyond the cardiovascular risks associated with chronic kidney disease and dialysis, kidney transplantation introduces unique risk factors, including immunosuppressive medication-induced post-transplant hyperglycemia, dyslipidemia, and hypertension. Additionally, the effects of suboptimal allograft function, such as chronic inflammation, oxidative stress, endothelial dysfunction, and chronic volume overload, as well as anemia, mineral bone disease, left ventricular hypertrophy, and homocysteinemia further heighten the risk of PTCVD [5,6,7]. Emerging evidence also suggests a role of adaptive cellular and humoral immunity in the development of CVD, with the expansion of pro-inflammatory and anti-apoptotic cytotoxic T-cells being associated with PTCVD [6]. Post-transplant DNA viremia such as infection with cytomegalovirus has also been shown to increase cardiovascular mortality by upregulating cytotoxic T-cells [6,7].

Cardiovascular disease accounts for one-third of all post-transplantation deaths, with the risk increasing significantly for individuals aged 50 years and above compared to the general population [8].

With the introduction of newer and more potent immunosuppressive agents, the interplay between traditional, chronic kidney disease (CKD)-related and transplant-specific risk factors in the genesis of cardiovascular complications warrants careful examination. Furthermore, despite evidence supporting cardiovascular risk modification through interventions such as blood pressure control, lipid modification, renin-angiotensin system blockage, and antiplatelet treatment, these strategies are often inadequately implemented in transplant recipients [4]. 

Against the backdrop of contemporary immunosuppressive strategies, this study aims to refine our understanding of PTCVD dynamics in KTRs without pre-existing CVD. Through the evaluation of a sizable cohort of KTRs spanning two decades, our aim is to unravel the nuanced interplay of factors contributing to the development of PTCVD and delineate its associations with graft outcomes and recipient mortality. 

In the evolving landscape of kidney transplantation, where the optimization of long-term outcomes has become imperative, insights derived from this study may guide clinicians in tailoring interventions to mitigate the burden of PTCVD, thereby advancing the holistic care of kidney transplant recipients.

In this context, we aimed to assess the cumulative incidence of CVD after transplantation and determine the risk factors of PTCVD in recipients devoid of a pre-existing diagnosis of CVD. Second, we assessed the impact of PTCVD on long-term graft survival and recipient survival.

## 2. Materials and Methods

### 2.1. Study Design

In this retrospective‚ single-center cohort study, patients who underwent kidney transplantation between January 2000 and December 2020 were included. To ensure a cohort without pre-existing cardiovascular disease, recipients with confirmed cardiovascular disease before kidney transplantation were excluded. Additionally, recipients with early graft failure (graft failure during the first three months post-transplantation) or loss to follow-up within three months of transplantation were excluded. 

### 2.2. Data Collection

Demographic, clinical, and laboratory data for each recipient were extracted from electronic patient records. Data were collected until one of the following clinical endpoints occurred: allograft loss, patient death, last reported contact with the center, or the conclusion of the study period (31 December 2020).

The study was conducted according to the principles of the Declaration of Helsinki. The study received written approval from the hospital Research Ethics Committee of the Northern Care Alliance Manchester (Reference code: S21HIP03). As this was an observational study with the complete anonymization of patient details and secondary use of real-world data, individual patient consent was not required.

### 2.3. Study Cohort

Participants had kidney transplantation surgeries at the Manchester Regional Transplant Center. Subsequently, recipients were typically transferred back to our renal center 3–4 months post-transplantation. In instances where recipients encountered early posttransplant complications requiring further intervention, such as acute rejection, ureteric stenosis, or allograft vascular stenosis, repatriation might be delayed beyond the fourth-month post-transplantation.

### 2.4. Explanatory Variables

Several key explanatory variables were considered in the analysis, encompassing age at transplantation, gender, ethnicity, body mass index (BMI), primary kidney disease, dialysis vintage, history of pre-transplant diabetes, type of allograft received, degree of HLA mismatch, immunosuppressive regimen, post-transplant diabetes, post-transplant DNA virus infections, and smoking history. Serial annual laboratory test values included tacrolimus level, parathyroid hormone (PTH) level, urine protein creatinine ratio (uPCR), and C-reactive protein (CRP). These were summarized to obtain the cohort median or mean values of the aggregated laboratory parameters measured over the total follow-up period. This provided a summary measure characterizing the central tendency of the aggregated laboratory test results for each participant. The baseline estimated glomerular filtration rate (eGFR), estimated at three months after transplantation, was defined using the modified Modification of Diet in Renal Disease (MDRD) equation [9]. Acute rejections were confirmed by kidney transplant biopsy, typically indicated by a deterioration in graft function and worsening proteinuria. The mean blood pressure was also recorded.

Variables were included to comprehensively capture and account for various demographic and clinical characteristics that could potentially influence or explain the observed outcomes of the study.

### 2.5. Outcome Variables

The study considered three primary outcome variables: time to PTCVD, death-censored graft survival (DCGS), and recipient survival. The rate of eGFR decline for each patient, defined as the slope of change in the eGFR, was determined through a linear mixed-effects model by regressing the yearly eGFR values on time (years) post-transplantation. This created a quantitative measure of the rate of decline of graft function over time for each recipient.

### 2.6. Post-Transplant Cardiovascular Disease Definition

PTCVD was defined as the presence of various cardiovascular conditions diagnosed after transplantation, including ischemic heart disease, myocardial infarction, chronic rhythm disturbances (such as atrial fibrillation or the need for a cardiac pacemaker), heart failure, cerebrovascular disease, peripheral vascular disease, and other myocardial and valvular heart diseases. The managing clinician performed the adjudication of cardiovascular events.

### 2.7. Pre-Transplant Cardiovascular Screening

Before transplantation, all transplant candidates are assessed for the presence and severity of CVD before listing for transplantation. This involves taking a comprehensive cardiovascular history, physical examination, a 12-lead electrocardiogram (ECG), and an echocardiogram. Candidates who are at high risk of CVD, patients aged 60 years or more, those who are diabetic, those with a pre-existing cardiovascular disease, and those with abnormal ECG or echocardiographic features or with poor functional capacity will go on to have non-invasive cardiac screening, usually in the form of a dobutamine stress echocardiogram (DSE). Candidates with ongoing signs or symptoms of CVD, those aged 60 years or more, and those with abnormal non-invasive cardiac screening tests are referred for a cardiology assessment as they need an invasive investigation and further management before transplantation. While on the kidney transplant waiting list, potential recipients undergo a repeat ECG and echocardiogram every two years and a DSE every three to four years unless there is a new abnormal finding on the echocardiogram. Patients on the simultaneous kidney-pancreas waiting list have yearly cardiac investigations. 

Patients with valvular heart disease are also referred for cardiology assessment as they need valvular surgery before being listed for transplantation. 

Through the screening of primary care and hospital records, patients without documented diagnoses of major adverse cardiovascular events (MACE) before screening, coupled with the absence of evidence of cardiovascular disease (CVD) after undergoing the aforementioned screening, are classified as not having clinically significant CVD. Additionally, new biomarker identification is also considered in this classification process.

### 2.8. Immunosuppression

All the recipients had maintenance immunosuppression with a calcineurin inhibitor (CNI)-based regimen, utilizing either tacrolimus or cyclosporin. Individuals without contraindications also received an anti-proliferative agent—either mycophenolic acid (MPA) or azathioprine (AZA). A tailored approach to corticosteroid administration was implemented according to the immunologic risk status of the recipients. Patients with a standard immunologic risk profile received a short course of corticosteroids lasting 1–2 weeks. Those deemed to be at high immunologic risk, characterized by factors such as younger recipients or older donors, a calculated panel reactive antibody (cPRA) exceeding 20%, the presence of a donor-specific antibody (DSA), or delayed onset of graft function, had an extended duration of corticosteroid maintenance.

A critical evaluation of corticosteroid treatment occurred at 3 and 6 months post-transplantation, where decisions regarding continuation or discontinuation were made based on the balance of the perceived risk of acute rejection and opportunistic infections.

### 2.9. Statistical Analysis

Descriptive statistics were presented as frequencies (percentages) for categorical data and mean ± standard deviations (SDs) or medians and interquartile ranges (IQR) for numeric data. For group comparison, the two-tailed unpaired t-test was used for parametric variables, Wilcoxon’s sign-rank test was used for non-parametric numeric variables, and Pearson’s chi-squared or Fisher’s exact test was used for categorical variables.

Fine and Gray’s competing risk regression model [10] was constructed to determine the cumulative incidence and independent predictors of PTCVD. The dependent variable against which each potential risk factor was examined was the time to cardiovascular disease diagnosis, measured in years. This method allows for the fact that recipients who die are no longer at risk of developing post-transplant CVD. This differs from the standard Kaplan–Meier model, which would introduce bias as it assumes that those who die remain at risk in the future. In the univariable analysis, covariates that demonstrated a correlation with time to PTCVD at a *p*-value of less than 0.20 were selected for inclusion in the multivariable competing risk regression model. We adopted a *p*-value threshold of less than 0.20 based on recommendations from previous studies, which suggested this was an appropriate criterion for variable inclusion [7]. All covariates meeting this threshold were subsequently incorporated into the multivariable model. Next, employing a stepwise backward elimination method based on the significance level of the variables, we iteratively removed variables with *p*-values greater than 0.10 from the model. This process resulted in the retention of only those variables with *p*-values less than or equal to 0.10 in the final multivariable model.

To evaluate the robustness of our approach, we conducted a sensitivity analysis using multivariable Cox regression on the aforementioned variables. We applied backward elimination methods based on the Akaike information criterion (AIC) to determine variable selection in this analysis.

Graft and recipient survival curves were constructed using the Kaplan–Meier method and compared using the log-rank test. The Cox proportional hazards model was used to examine the effect of PTCVD on death-censored graft loss (DCGL) and recipient death, respectively while adjusting for factors known from the literature to influence graft and recipient outcomes.

In the analysis of death-censored graft survival, the following variables were considered potential confounders: age, primary renal disease, donor type, total HLA mismatch, baseline eGFR, history of acute rejection, and number of immunosuppressive agents. The potential confounders that were considered in the analysis of recipient death included age, primary renal disease, donor type, total HLA mismatch, baseline eGFR, history of acute rejection, and smoking history. These potential confounders were chosen based on previous knowledge [11,12,13].

The level of significance was set at *p* < 0.05. Regression analyses were conducted with the case-wise deletion of observations with missing data. All analyses were performed using R statistical software version 4.3.0 (R Core Team, 2021) [14].

## 3. Results

**Figure 1:**
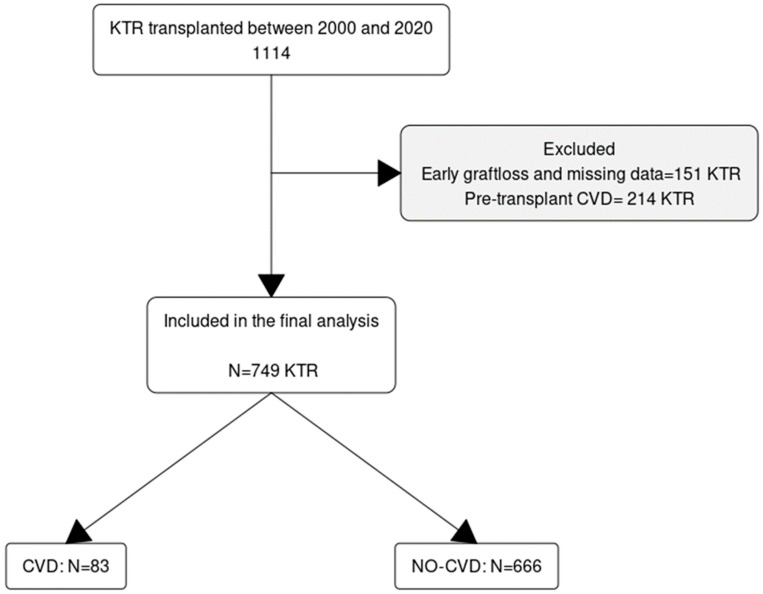
Flowchart of subject selection for the study.

A total of 1114 KTRs who underwent transplantation between January 2000 and December 2020 were evaluated; 365 recipients who did not meet the inclusion criteria, including 214 (19%) patients with prior CVD, were excluded from the final analysis, leaving 749 recipients for complete analysis. The flow chart of patient selection is shown in Figure 1, and the baseline characteristics of the participants are summarized in Table 1. The cohort was followed up for a total of 6017 person-years, and the median follow-up was 8.1 (IQR 4.5–13.0) years. The mean age at transplantation was 45 ± 15 years; 40% were female, and 81% were of White ethnicity. Pre-emptive transplantation was performed in 32% of recipients, while those without pre-emptive transplants had a median duration on dialysis of 26 months. The most common etiology of chronic kidney disease (CKD) was glomerulonephritis (GN). Approximately 30% of recipients received an allograft from a living donor. Most (90%) recipients received tacrolimus-based maintenance immunosuppression, and 46% had more than six months of corticosteroid therapy. The median baseline eGFR was 51 (IQR 41–64 mL/min/1.73m^2^.

After transplantation, 83 (11%) recipients experienced PTCVD, 69 (9%) recipients experienced graft loss, and 115 (15%) recipients died.

**Figure 2:**
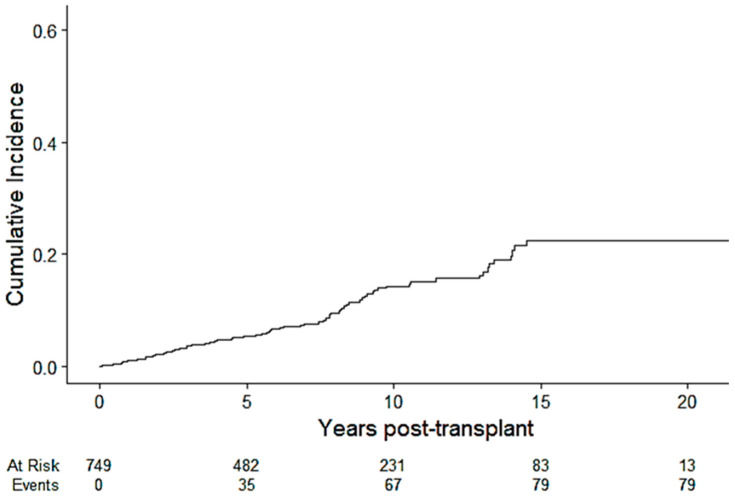
Cumulative incidence of PTCVD over the follow-up period.

The overall cumulative incidence of PTCVD at 1, 5, 10 and 20 years was 1.1% (0.51%, 2.0%), 5.4% (3.8%, 7.3%), 14.3% (11.1%, 17.9%), and 22.5% (17.2%, 28.2%), respectively (Figure 2). The median time to CV events was 5.8 (IQR 2.5–6.2) years, and the median CV event-free survival was 7.6 (IQR 4.1–12) years. The incidence rate of CVD per 1000 person-years was 13.7 (i.e., 1.37% per year).

The PTCVD patients were older at transplantation (50 vs. 44.5 years; *p* < 0.001) and more likely to have received corticosteroids for greater than 6 months (60.2% vs. 44.2%; *p* = 0.020). The PTCVD group had a lower baseline eGFR (median eGFR:46 vs. 52 mL/min), a lower average tacrolimus level (median: 4.6 vs. 5.7 ug/L; *p* = 0.003), and a lower mean hemoglobin level (mean:121.3 vs. 126.7 g/L; *p* = 0.023). The PTCVD group also exhibited a higher median parathyroid hormone (PTH) level (13.1 vs. 10.0 ng/L; *p* = 0.009) and a higher median urinary protein-to-creatinine ratio (uPCR) (40.5 vs. 22.7 mg/mmol; *p* = 0.024).

Conversely, factors such as sex, ethnicity, primary renal disease, number of HLA mismatches, history of acute rejection, DNA viremia, and smoking history were comparable between the PTCVD and non-PTCVD groups.

### 3.1. Predictors of PTCVD

Table 2 presents both the univariable and multivariable competing risk regression models assessing the risk factors associated with the development of PTCVD. In the univariable competing risk model, considering graft loss and recipient death as competing risks, recipient age, duration of dialysis vintage (per year), and the slope of estimated glomerular filtration rate (eGFR) decline exhibited significant associations with PTCVD. In the multivariable model, the independent predictors of PTCVD included an increase in recipient age (sub-hazard ratio [SHR]: 1.22; 95% CI: 1.01–1.46; *p* = 0.036) per year, duration of dialysis (SHR: 1.07; 95% CI: 1.00–1.14; *p* = 0.048) for each year spent on dialysis, and the slope of eGFR decline (SHR: 0.91; 95% CI: 0.86–0.98; *p* = 0.007) per mL/min/yr increase. Also, a higher baseline eGFR exhibited an increased protective effect (SHR, 0.98; 95% CI; 0.96–1.00; *p* = 0.032) per 10 mL/min/1.73m^2^ increase.

We conducted a sensitivity analysis to test the robustness of the competing risk regression analysis. In this analysis, we conducted a Cox regression analysis as documented in the Section 2. The results are shown in Supplementary Tables S1 and S2. The outcome of this sensitivity analysis did not substantially differ from that when using our initial method. 

### 3.2. Graft and Recipient Survival

The median survival time for the cohort was >20 years (IQR; 13–20 years). The median survival for those with PTCVD was 15 years (IQR; 9–20 years), whereas those without PTCVD had a median survival of >20 years (IQR; 14–20 years).

Over the observation period, 69 recipients (9.2%) lost their graft—13 (15.7%) from the PTCVD group and 56 (8.4%) from the No-PTCVD group. 132 (17.6%) recipients died with a functioning graft—31 (37.3%) in the PTCVD group vs. 101 (15.2%) in the No-PTCVD group. 

**Figure 3:**
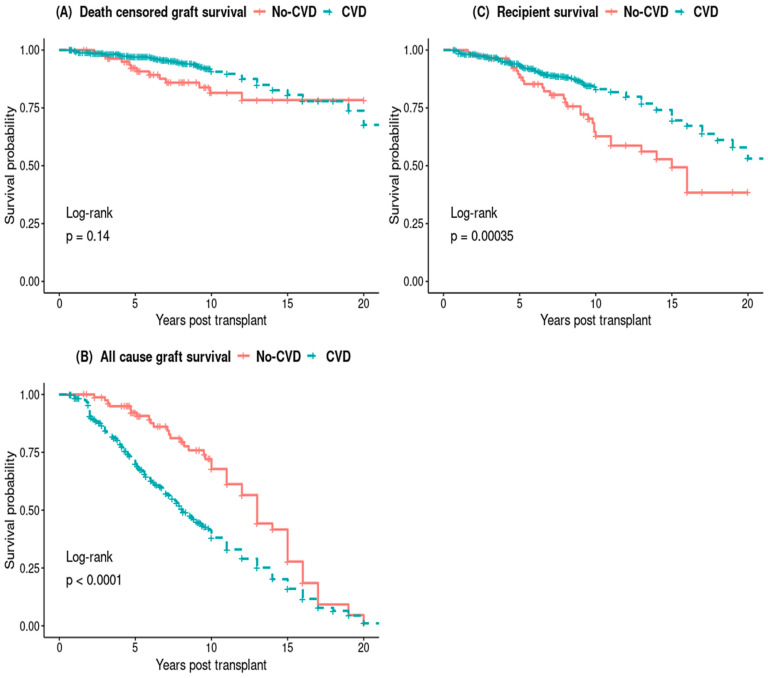
Kaplan–Meier survival curves of death censored graft survival (**A**) and all-cause graft survival (**B**) and recipient survival (**C**).

The Kaplan–Meier survival curves did not show any difference in death-censored graft survival between the PTCVD and No-PTCVD groups, while all-cause graft survival and recipient survival was significantly lower in those with PTCVD (Figure 3). 

**Figure 4:**
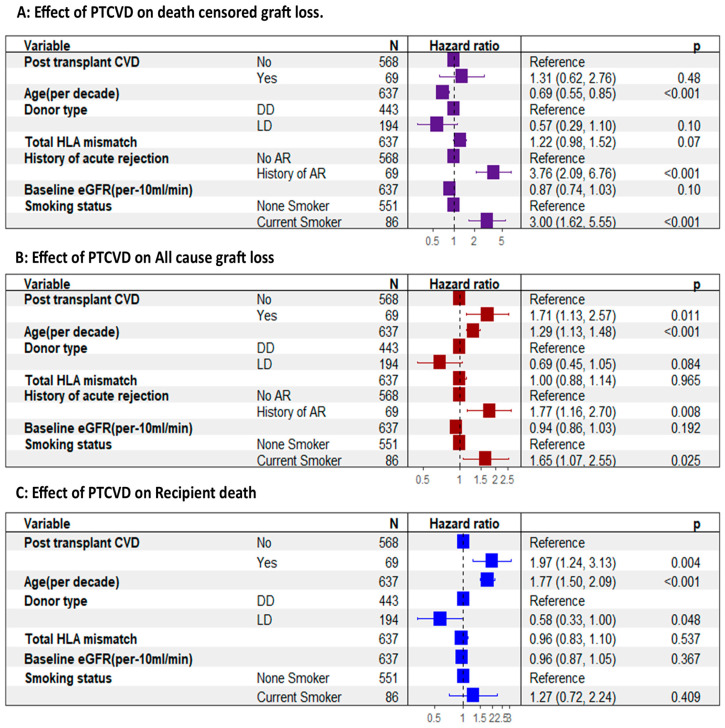
Effect of PTCVD on graft and recipient outcomes adjusted for confounding factors. (**A**) showing no difference in death-censored graft loss between those with PTCVD and those without. (**B**) shows a significantly higher all-cause graft loss associated with PTCVD. (**C**) shows a significantly higher recipient death amongst those who experience PT CVD. AR, acute rejection; DD, deceased donor; LD, living donor.

The adjusted multivariable Cox proportional hazard model showed no significant difference in the DCGL between the two groups, but there was a notable 71% increase in the relative hazard of all-cause graft loss in the PTCVD group (adjusted hazard ratio [aHR], 1.71; 95% CI, 1.13–2.57; *p* < 0.001) (Figure 4B). Similarly, the relative hazard of recipient death was 97% higher in the PTCVD group (aHR, 1.97; 95% CI, 1.24–3.13; *p* = 0.004) (Figure 4C).

## 4. Discussion

KTRs navigate a distinctive array of health challenges, particularly in relation to the prevention and management of CVD. As highlighted by previous research, KTRs already contend with a considerable burden of CVD risk factors [15,16,17]. While KTRs generally present a lower CVD risk than individuals with end-stage kidney disease (ESKD) awaiting transplantation, their cardiovascular risk remains significantly elevated—it is up to 50 times greater than that of their age-matched counterparts in the general population [17,18].

Our study’s findings offer crucial insights into the prevalence, predictors, and outcomes of PTCVD. 

The cumulative incidence of PTCVD, as revealed in our study, aligns with previous reports, acknowledging potential variations arising from differences in CVD definition and cohort characteristics [19,20,21]. Notably, our cohort’s exclusion of recipients with a pre-transplant CVD history distinguishes it from some earlier studies.

The study identified age at transplantation, duration on dialysis, baseline eGFR, and the rate of post-transplant eGFR decline as independent predictors of PTCVD. These findings collectively underscore the enduring influence of CKD-related factors in predisposing recipients to PTCVD. Intriguingly, traditional CVD risk factors, such as smoking, gender, and diabetes, did not exhibit significant associations with PTCVD in our study. This implies that PTCVD may be more strongly linked to factors stemming from impaired kidney function rather than traditional cardiovascular risk factors. 

Furthermore, the absence of a significant impact of the immunosuppressive regimen type on PTCVD risk is somewhat reassuring. The reasons for this are not entirely clear, and the lack of recorded data on steroid doses in the cohort adds to the complexity. Despite this limitation, it is likely that recipients were not exposed to high doses of maintenance corticosteroids. In addition, the study’s findings indicate that median tacrolimus levels were not notably elevated at 5.5 ± 2.7 mmol/L. 

Immunosuppressive agents exert direct effects on the cardiovascular system. These include left ventricular hypertrophy (LVH), myocardial fibrosis, arrhythmia, hypertension, dyslipidemia, and coronary atherosclerosis. CNIs have been associated with cardiac hypertrophy. CNIs promote the transcription of genes, which increase left ventricular mass through CNI-induced increases in fibrosis and collagen deposition [22]. Furthermore, CNI and corticosteroids are associated with an increased risk of hypertension, hyperlipidemia, and vascular remodeling. On the other hand, mTOR inhibitors have been suggested to reduce cardiac hypertrophy by attenuating cardiac remodeling and reducing cardiac fibroblast proliferation and collagen secretion. There is currently no evidence to suggest that antiproliferative agents have any direct effects on cardiac myocytes [22].

The considerable impact of PTCVD on recipient survival, marked by an 85% increase in the relative risk of death, is consistent with findings in the existing literature. However, the absence of a significant difference in death-censored graft loss between recipients with and without PTCVD in our study deviates from some prior reports [23]. However, this is corroborated by supporting evidence in other studies [20]. This inconsistency could be attributed to the observed tendency of recipients with PTCVD to succumb to cardiovascular events before experiencing graft failure.

The findings of this study emphasize the critical role of risk assessment, early detection, and preventive interventions in mitigating post-transplant cardiovascular disease (PTCVD) risk among kidney transplant recipients. Pre-transplant CVD screening was intended to detect at-risk patients and prevent PTCVD onset by facilitating risk stratification, early detection, and proactive intervention [24,25]. However, there is a lack of evidence supporting the effectiveness of pre-transplant screening in conferring survival benefits during the post-transplant period [24,25]. Current evidence suggests that some screening procedures may not offer clear benefits and may even pose potential risks to patients [24,25]. Non-invasive testing such as myocardial perfusion scans, stress echocardiograms, and computed tomography coronary angiograms are popular screening methods for the initial screening for CVD, but the accuracy of non-invasive screening remains a subject of debate. This is mainly due to the differences in the pathophysiology of coronary artery disease in patients with CKD compared to non-CKD patients [26]. Moreover, a positive non-invasive test for early post-transplant cardiovascular disease may not necessarily imply good discrimination for prognostically significant CVD [27]. While coronary angiography remains the best predictor of cardiovascular adverse events in transplant recipients, its use is associated with an increased risk of complications, including those related to iodinated contrast media, as well as complications due to the invasive nature of the procedure [26]. Therefore, there is a pressing need for further research to understand better the role and effectiveness of pre-transplant CVD screening in kidney transplant recipients.

Efforts should be directed toward developing safer and more accurate screening methods tailored to the unique characteristics and needs of CKD patients and transplant recipients. Biomarker development in this area, including the utility of established cardiac biomarkers such as cardiac troponin, NTProBNP, and placental growth factor (PLGF) before and after transplantation, holds promise. Furthermore, the identification of newer biomarker may contribute to the accuracy of imaging and clinical assessments, thereby enhancing the effectiveness of both pre-and post-transplant screening strategies. Enhancing risk assessment and management strategies for this vulnerable patient population is essential for improving long-term transplant outcomes and patient well-being.

Despite its contributions, our study has limitations inherent to its retrospective design, which may introduce potential biases such as selection bias, confounding, misclassification, and incomplete data. While we assumed no pre-transplant CVD in our evaluated cohort based on recorded data and robust pre-transplant screening, may not be entirely accurate due to undiagnosed cases of pre-transplant CVD.

A major limitation was that certain traditional cardiovascular risk factors, such as hypertension and dyslipidemia, were not included in the predictive model. The complex nature of blood pressure fluctuations pre- and post-transplantation, the non-protocolled measurement of lipid profiles only in certain patients in the post-transplant period, and the variable use of statin therapy posed challenges in accurately quantifying their effects on CVD risk. Due to the retrospective nature of this real-world study, including the above factors would have introduced a potential for selection bias, as patients with CVD risk factors or symptoms were more likely to have a lipid profile assay than those without. Similarly, patients at higher risk of CVD were more likely to have statins prescribed.

Lastly, it is worth noting that a considerable number of recipients underwent modifications to their immunosuppression regimen throughout the follow-up period. These alterations might have been prompted by complications such as infections, acute rejection, and medication side effects. Consequently, accurately delineating the impact of their immunosuppressive regimen on PTCVD over the follow-up duration posed a considerable challenge. Despite these limitations, our study aimed to uncover novel post-transplant CVD risk predictors beyond established factors. We acknowledge the need for future research to address the limitations identified in our study. Prospective studies capturing comprehensive data on established risk factors and treatment variables will be essential to elucidate further the complex relationship between these factors and post-transplant CVD risk.

## 5. Conclusions

In conclusion, our study provides valuable insights into the predictors and outcomes of PTCVD, emphasizing the influence of chronic kidney disease-related factors, such as duration on dialysis, baseline eGFR, and the rate of eGFR decline, in the development of PTCVD. The substantial impact of PTCVD on recipient survival underscores the necessity for a nuanced risk management approach that incorporates strategies to mitigate factors associated with kidney function deterioration. Overall, our findings further enrich the understanding of the complex dynamics surrounding cardiovascular health in kidney transplant recipients.

## Figures and Tables

**Table 1:** Clinical characteristics of the participants according to the history of PTCVD.

	PTCVD (N = 83)	No-PTCVD (N = 666)	Total (N = 749)	*p* Value
**Age (years)**				0.001
- Mean (SD)	50.1 (13.6)	44.5 (15.3)	45.1 (15.2)	
**Sex**				0.565
- Male	47 (56.6%)	399 (59.9%)	446 (59.5%)	
- Female	36 (43.4%)	267 (40.1%)	303 (40.5%)	
**Ethnicity**				0.426
- White	68 (81.9%)	542 (81.4%)	610 (81.4%)	
- Asian	13 (15.7%)	91 (13.7%)	104 (13.9%)	
- Black	2 (2.4%)	13 (2.0%)	15 (2.0%)	
- Other	0 (0.0%)	20 (3.0%)	20 (2.7%)	
**Primary renal disease**				0.090
- ADPKD	6 (7.2%)	79 (11.9%)	85 (11.3%)	
- GN	30 (36.1%)	190 (28.5%)	220 (29.4%)	
- DKD	2 (2.4%)	71 (10.7%)	73 (9.7%)	
- HKD	7 (8.4%)	41 (6.2%)	48 (6.4%)	
- Reflux/CPN	17 (20.5%)	105 (15.8%)	122 (16.3%)	
- Unknown	10 (12.0%)	109 (16.4%)	119 (15.9%)	
- Other	11 (13.3%)	71 (10.7%)	82 (10.9%)	
**Pre-transplant diabetes**				0.055
- No	77 (92.8%)	566 (85.0%)	643 (85.8%)	
- Yes	6 (7.2%)	100 (15.0%)	106 (14.2%)	
**Transplant number**				0.872
- Mean (SD)	1.2 (0.4)	1.1 (0.4)	1.1 (0.4)	
**Pre-emptive transplant**				0.069
- No	61 (77.2%)	429 (67.1%)	490 (68.2%)	
- Yes	18 (22.8%)	210 (32.9%)	228 (31.8%)	
**Donor type**				0.224
- DD	63 (75.9%)	461 (69.4%)	524 (70.1%)	
- LD	20 (24.1%)	203 (30.6%)	223 (29.9%)	
**Total HLA mismatch**				0.460
**-** Mean (SD)	2.33 (1.47)	2.46 (1.4)	2.44 (1.4)	
**Total ischaemia time**				0.115
**-** Mean (SD)	14.0 (7.5)	12.5 (7.7)	12.7 (7.7)	
**Main immunosuppression**				0.005
- MTORI	1 (1.2%)	10 (1.5%)	11 (1.5%)	
- Cyc	15 (18.3%)	50 (7.6%)	65 (8.8%)	
- Tac	66 (80.5%)	599 (90.9%)	665 (89.7%)	
**Antimetabolite**				0.018
- None	16 (19.8%)	64 (9.7%)	80 (10.8%)	
- MPA	53 (65.4%)	506 (76.9%)	559 (75.6%)	
- Aza	12 (14.8%)	88 (13.4%)	100 (13.5%)	
**Steroid maintenance**				0.020
- <2 wks	32 (38.6%)	352 (53.3%)	384 (51.6%)	
- 2 w–6mo	1 (1.2%)	17 (2.6%)	18 (2.4%)	
- >6mo	50 (60.2%)	292 (44.2%)	342 (46.0%)	
**Number of immunosuppressive agents**				0.026
- Single Agent	7 (8.4%)	26 (4.0%)	33 (4.5%)	
- Two Agents	38 (45.8%)	389 (59.4%)	427 (57.9%)	
- Three Agents	38 (45.8%)	240 (36.6%)	278 (37.7%)	
**Donor CMV status**				0.632
**-** Positive	40 (58.0%)	301 (54.9%)	341 (55.3%)	
**Recipient CMV status**				0.106
- Positive	44 (64.7%)	298 (54.4%)	342 (55.5%)	
**Smoking history**				0.775
- Never smoked	48 (64.9%)	408 (66.7%)	456 (66.5%)	
- Current smoker	12 (16.2%)	81 (13.2%)	93 (13.6%)	
- Ex-smoker	14 (18.9%)	123 (20.1%)	137 (20.0%)	
**History of acute rejection**				0.259
- No AR	70 (84.3%)	590 (88.6%)	660 (88.1%)	
- History of AR	13 (15.7%)	76 (11.4%)	89 (11.9%)	
**Duration of RRT (mo)**				0.069
- Median (IQR)	30.0 (14.858)	26.0 (12.045)	26.0 (12.048)	
**Pre-transplant BMI (kg/m^2^)**				0.519
- Mean (SD)	27.4 (4.8)	26.7 (8.4)	26.8 (8.1)	
**Post-transplant diabetes**				0.053
- Yes	19 (22.9%)	98 (14.7%)	117 (15.6%)	
**Baseline eGFR(mL/min/1/73m^2^)**				0.030
- Median (IQR)	46.0 (37.0–61.0)	51.5 (41.0–64.3)	51.0 (40.0–64.0)	
**CMV { XE “CMV” } viremia**				0.395
- Yes	9 (10.8%)	95 (14.3%)	104 (13.9%)	
**EBV { XE “EBV” } viremia**				0.342
- Yes	13 (15.7%)	80 (12.0%)	93 (12.4%)	
**Polyoma viremia**				0.131
- Yes	68 (10.2%)	13 (15.7%)	81 (10.8%)	
**Any DNA virus infection**				0.204
- Yes	29 (34.9%)	188 (28.2%)	217 (29.0%)	
**Average tacrolimus level (mmol/L)**				0.003
**-** Mean (SD)	4.6 (2.2)	5.7 (2.7)	5.5 (2.7)	
**Mean haemoglobin (g/L)**				0.023
**-** Mean (SD)	121.3 (21.4)	126.7 (19.4)	126.1 (19.8)	
**Mean uPCR (mg/mmol)**				0.024
**-** Median (IQR)	40.5 (13.2–1.0)	22.7 (11.3–63.3)	24.3 (11.4–66.5)	
**Average PTH { XE “PTH” } level (ng/L)**				0.009
- Median (IQR)	13.1 (8.2–18.1)	10.0 (6.7–15.5)	10.2 (6.8–16.2)	
**eGFR slope (mL/min/year)**				0.079
**-** Median (IQR)	−1.33(−3.21–0.23)	−0.77(−2.60–0.56)	−0.81(−2.66–0.55)	

Data are shown as mean ± SD, median (interquartile range), or number (percentage). ADPKD, Autosomal dominant polycystic kidney disease; AR, acute rejection; Aza, azathioprine; BMI, body mass index; CMV, Cytomegalovirus; CPN, chronic pyelonephritis; CVD, cardiovascular disease; Cyc, cyclosporin; DKD, diabetic kidney disease; DD, deceased donor; eGFR, estimated glomerular filtration rate; GN, glomerulonephritis; HKD, hypertensive kidney disease; LD, living donor; HLA, human leucocyte antigen; MPA, mycophenolic acid; MTORI, mammalian target of rapamycin inhibitor; PTH, parathyroid hormone.

**Table 2:** Univariable and multivariable competing risk regression models showing the predictors of time to PTCVD.

	Univariate Competing Risk Regression Model			Multivariate Competing Risk Regression Model		
Characteristic	SHR	95% CI	*p*-Value	SHR	95% CI	*p*-Value
Recipient age at transplant (per decade)	1.26	1.10–1.45	<0.001	1.22	1.01–1.46	0.036
Female	1.06	0.68–1.63	0.8			
Ethnicity						
White	1.00	—	—			
Asian	1.28	0.69–2.36	0.43			
Black	1.70	0.44–6.60	0.44			
Other	0.00	0.00–0.00	<0.001			
Pre-transplant BMI (per 5 kg/m^2^)	1.07	1.00–1.15	0.065			
Primary renal disease						
ADPKD{ XE “ADPKD” }	1.00	—	—			
GN{ XE “GN” }	1.48	0.61–3.56	0.39			
DKD{ XE “DKD” }	0.38	0.08–1.85	0.23			
HKD{ XE “HKD” }	2.15	0.72–6.39	0.17			
Reflux/CPN{ XE “CPN” }	1.58	0.63–3.95	0.33			
Unknown	0.95	0.35–2.59	0.92			
Other	1.46	0.53–4.00	0.46			
Pre-emptive transplant	0.65	0.39–1.09	0.11			
Dialysis vintage (per year)	1.06	1.00–1.12	0.037	1.07	1.00–1.14	0.048
Pre-transplant diabetes	0.56	0.25–1.28	0.17			
Donor type						
DD{ XE “DD” }	1.00	—	—			
LD{ XE “LD” }	0.78	0.47–1.30	0.34			
Total mismatch	1.03	0.88–1.22	0.71			
Total ischaemic time	1.01	0.99–1.04	0.34			
CNI{ XE “CNI” }						
Tacrolimus	1.00	—	—			
Cyclosporine	1.57	0.90–2.75	0.11			
Antimetabolite						
None	1.00	—				
MPA{ XE “MPA” }	0.83	0.47–1.46	0.52			
Aza{ XE “Aza” }	0.72	0.33–1.56	0.41			
Corticosteroid treatment						
<2 wks	1.00	—	—			
2 w–6 mo	0.85	0.11–6.62	0.88			
>6 mo	1.26	0.80–1.97	0.32			
Number of immunosuppressive agents	1.16	0.79–1.68	0.45			
Recipient CMV-positive	1.53	0.93–2.52	0.09			
Donor CMV-positive	1.08	0.66–1.76	0.76			
CMV viremia	0.76	0.39–1.50	0.44			
EBV viremia	1.01	0.56–1.81	0.98			
Polyoma viremia	1.80	0.98–3.29	0.057			
Smoking history						
Never smoked	1.00	—				
Current smoker	1.31	0.72–2.40	0.37			
Ex-smoker	1.17	0.63–2.19	0.62			
Median tacrolimus level	0.91	0.82–1.00	0.061	0.93	0.85–1.02	0.10
Post-transplant diabetes	1.55	0.93–2.58	0.092			
Baseline eGFR (per 10 mL/min increase)	0.91	0.79–1.03	0.14	0.98	0.96–1.00	0.032
Slope of eGFR	0.96	0.92, 1.00	0.073	0.91	0.86, 0.98	0.007
Average uPCR	1.00	1.00–1.00	0.12			
Average PTH (per 10 units increase)	1.04	0.98–1.10	0.18			

Competing risk regression showing factors associated with time to PTCVD, with death as the competing risk; SHR, sub-hazard ratio; Variables with *p*-values less than 0.20 in univariable analysis were included in the multivariable model. Continuous variables missing for more than 10% of the population were excluded from multivariable analyses. Significant variables at *p* < 0.20 included age at transplantation, ethnicity, pre-transplant BMI, pre-emptive transplant, duration of RRT, type of CNI, recipient CMV status, polyoma viremia, median tacrolimus level, baseline eGFR, mean UPCR, and median parathyroid hormone level. A stepwise backward elimination method was employed in multivariable analysis, retaining variables with *p*-values less than or equal to 0.10. ADPKD, adult polycystic kidney disease; DKD, diabetic kidney disease; HKD, hypertensive kidney disease; CMV; cytomegalovirus; CI confidence interval; CPN, chronic pyelonephritis; DD, deceased donor; LD, living donor; CNI, calcineurin inhibitor; MPA, mycophenolic acid; Aza, azathioprine; ISA, immunosuppressive agents; NODAT, new-onset diabetes after transplant; PTH; parathyroid hormone; uPCR, urine protein/creatinine ratio.

## Data Availability

The data presented in this study are available on request from the corresponding author (Chukwuma Austin Chukwu).

## Supplementary Materials

The following supporting information can be downloaded at: https://www.mdpi.com/article/10.3390/jcm13102734/s1, Table S1: Summary of variable selection process through backward elimination using AIC. Table S2: Summary of predictors of time to CVD retained in the multivariable Cox regression model after backward elimination based on variable AIC.
