# Supplementary material for: Post-Transplant Cardiovascular Disease in Kidney Transplant Recipients: Incidence, Risk Factors, and Outcomes in the Era of Modern Immunosuppression
Source: J Clin Med. 2024 May 7;13(10):2734. doi: 10.3390/jcm13102734 (PMC11122649; doi:10.3390/jcm13102734)
Supplement: Supplementary file 1 [file jcm-13-02734-s001.zip › jcm-2957874-supplementary.pdf]

Supplementary data

Supplementary table 1: Summary of variable selection process through backward elimination using AIC (variables eliminated from the model)

Step	Excluded variables	Number_parms	AIC
1		16	566.039128
2	ethnicity	13	555.012251
3	CNI type	12	550.91766
4	Mean uPCR	11	547.064139
5	polyoma	10	543.433676
6	Recipient CMV status	9	540.028482
7	Post transplant diabetes	8	536.811195
8	Recipient BMI	7	533.801781
9	Parathyroid hormone level	6	531.559514
10	preemptive_ transplant	5	529.668123

Variables excluded from the cox model based on the AIC; AIC, Akaike information criterion; BMI, Body mass index; CNI, calcineurin inhibitor; CMV, cytomegalovirus; uPCR, urine protein-creatinine ratio.

Supplementary table 2: Summary of predictors of time to CVD using multivariable Cox regression model with backward elimination based on variable AIC .

Variable	coefficient	aHR	95%CI	p-value
Recipient Age	0.04	1.04	(1.01-1.05)	**0.003**
Dialysis vintage	0.08	1.08	(1.02-1.15)	**0.006**
Median tacrolimus level	-0.15	0.86	(0.74-0.98)	**0.034**
Baseline eGFR (per 10ml/min/1.73^2)	-0.16	0.86	(0.73-1.01)	0.063
eGFR slope	-0.13	0.88	(0.80-0.95)	**0.003**

Variables retained in the Cox model after backward elimination of variables with high AIC. Included in the model are recipients' age, ethnicity, CNI type, mean urine protein creatinine ratio, polyoma viremia, recipient CMV status, history of pot transplant diabetes, Pretransplant BMI, median parathyroid hormone level, pre-emptive transplantation, dialysis vintage, median tacrolimus level, baseline eGFR and slope of eGFR. AIC, Akaike information criterion; aHR,

adjusted hazard ratio; 95% CI, 95% confidence interval. eGFR estimated glomerular filtration rate,
